# Lymphocintilographic evaluation of lymphatic circulation in victims of circuferential degloving injuries of the lower limbs

**DOI:** 10.1590/0100-6991e-20222981

**Published:** 2022-02-18

**Authors:** DANIEL FRANCISCO MELLO, JOSE CESAR ASSEF, AMERICO HELENE-JR

**Affiliations:** 1 - Irmandade da Santa Casa de Misericórdia de São Paulo, Serviço de Cirurgia Plástica - São Paulo - SP - Brasil; 2 - Irmandade da Santa Casa de Misericórdia de São Paulo, Serviço de Emergência - São Paulo - SP - Brasil

**Keywords:** Lymphedema, Soft Tissue Injuries, Skin, Fascia, Radionuclide Imaging, Linfedema, Lesões dos Tecidos Moles, Pé, Fáscia, Cintilografia

## Abstract

**Introduction:**

secondary forms of lymphedema may occur as consequence of tumors, surgeries, radiotherapy, trauma and infections. Degloving injuries are severe and infrequent forms of trauma, with avulsion at the level of muscular fascia, and consequent injury of the lymphatic system.

**Objective:**

to evaluate the alterations in lymphatic circulation in patients being victims of circumferential degloving injuries in the lower limbs, using lymphoscintigraphic*.*

**Patients and Methods:**

retrospective analysis of the cases treated in the period from 2010 to 2016. Segmental, circumferential and unilateral injuries with involvement of the lower limbs were included. Lymphoscintigraphy was performed after a minimum interval of 12 months after the end of treatment. The non-injured lower limb was used as control. The Kleinhans Semiquantitative Index (KSI) was used for the semiquantitative evaluation of the lymphoscintigraphic findings.

**Results:**

eighteen patients were evaluated, six of whom were female and 12 were male. The mean age was 28.11 years. The average vertical extension of the circumferential traumatized segment was 29.33cm. The injured area presented variations of 5 to 15% of the body surface, with an average of 8.95%. Lymphoscintigraphy was performed after an average interval of 22.55 months. Alterations were observed on the traumatized limb (TL) in 13 patients. All control limbs (CL) were normal. The mean KSI observed in TL was 8.32, while in CL, the average value was 0.58 (p<0.001)*.*

**Conclusion:**

patients with circumferential degloving injuries in the lower limbs present compromised lymphatic circulation and high probability to develop lymphedema.

## RESUMO

**Introdução:**

as formas secundárias de linfedema podem ocorrer em consequência de tumores, cirurgias, radioterapia, traumas e infecções. Os desenluvamentos são formas graves e infrequentes de trauma, sendo observada avulsão no plano da fáscia muscular, com consequente lesão do sistema linfático.

**Objetivo:**

avaliar as alterações na circulação linfática nos pacientes vítimas de desenluvamentos circunferenciais nos membros inferiores, a partir da análise linfocintilográfica.

**Pacientes e Métodos:**

análise retrospectiva dos casos atendidos no período de 2010 a 2016. Foram incluídos os casos com acometimento segmentar, circunferencial e unilateral em membros inferiores. A linfocintilografia foi realizada após um intervalo mínimo de 12 meses do término do tratamento. O membro inferior não traumatizado foi utilizado como controle. Para a avaliação semiquantitativa dos achados linfocintilográficos, utilizou-se o Índice de Kleinhans (ISQTK).

**Resultados:**

foram avaliados 18 pacientes, sendo seis do gênero feminino e 12 do masculino. A média de idade foi de 28,11 anos. A extensão vertical do segmento traumatizado circunferencial foi em média de 29,33cm. O segmento traumatizado apresentou variações de 5 a 15% da superfície corporal, com média de 8,95%. A linfocintilografia foi realizada após intervalo médio de 22,55 meses. Foram observadas alterações no exame linfocintilográfico do membro traumatizado (MT) em 13 pacientes. Todos os exames dos membros controle (MC) foram normais. O ISQTK médio observado no MT foi 8,32 (0,3-20,75). No MC o valor médio foi de 0,58 (0,15-0,75).

**Conclusão:**

os pacientes com desenluvamentos circunferenciais nos membros inferiores apresentam circulação linfática comprometida e alta probabilidade para o desenvolvimento de linfedema.

## INTRODUCTION

The lymphatic system is a complex network, made up of capillaries and lymphatic vessels, lymph nodes, and lymphatic organs, which are vitally important for the maintenance of liquid homeostasis and for immunological aspects^1^.

Lymphedema can be understood as a manifestation of the insufficiency of the lymphatic system due to an alteration in circulation, that is, a reduction in transport of primary or secondary origin. It should not be understood only as a simple local edema process, but as a complex and systemic disease^2^ ^,^ ^3^.

Secondary forms of lymphedema, also called obstructive, are the most frequent, in more than 95% of cases^4^ ^,^ ^5^. There are clinical signs, which are usually more accentuated the greater the degree of stasis and the longer the duration of the process.

The etiologies normally associated with this type of injury are^6^ ^,^ ^7^:

- Malignant neoplasms, as well as related treatments
- Infections and parasitic infestations
- Surgical trauma
- Radiotherapy
- Trauma

So far, there are no factors that allow predicting the occurrence of lymphedema after trauma, operations, or procedures, and genetic predispositions associated with environmental factors may play a role^3^ ^,^ ^8^ ^,^ ^9^.

There is scarce literature related to cases of post-traumatic lymphedema, and occurrences related to extensive injuries and deep burns can be found, with or without skin grafts, including prolonged latent periods^10^ ^-^ ^16^.

Some authors have described that post-traumatic lymphedema can occur months or even years after trauma to the lower limbs. They also highlighted that there are still significant limitations in analyzing the evolution of the lymphatic system in the healing process. They considered that there may be recanalization or even regeneration in scarring areas, including partial thickness skin grafts^15^ ^,^ ^17^ ^,^ ^18^.

Degloving injuries are uncommon skin and soft tissue traumas, characterized by avulsions of the skin and subcutaneous tissue from the plane of the muscle fascia, involving injury to the fasciocutaneous and musculocutaneous perforating vessels. They result from the application of sudden and high intensity forces with tangential vectors, promoting compression, stretching, torsion, and friction of the structures. Most injuries present as a continuity solution of variable size, with exposure of the fascia and muscles, being called open, typical, or anatomical degloving^19^ ^,^ ^20^.

In our service, we observed that, in cases with circumferential involvement, especially when there is greater vertical extension, postoperative edema of the distal segment was more significant and prolonged. This study aims to evaluate the sequelae and alterations of the lymphatic drainage in victims of circumferential degloving injuries of the lower limbs, based on lymphoscintigraphic analysis.

## METHODS

We conducted a retrospective analysis of medical and photographic records of degloving injuries cases affecting the lower limbs, treated in the period from 2010 to 2016.

We included cases with exclusive trauma to the lower limbs, with segmental, circumferential, and unilateral involvement, who underwent debridement of traumatized tissues, subsequent wound preparation, and partial skin grafting. 

The exclusion criteria were:

- Bilateral lower limb trauma;
- Associated vascular trauma;
- Amputation of lower limb segment;
- Previous edema of systemic etiology - heart, renal, thyroid, or liver failure;
- Personal history of vascular surgeries in the lower limbs;
- Personal history of thromboembolic events or diagnosis of post-thrombotic syndrome; and
- Morbid obesity.

We analyzed sex, age, date of trauma, interval until the lymphoscintigraphy examination, affected body surface (ABS) by degloving injury (in %, using the Lund and Browder table), and vertical extension of the traumatized circumferential segment (in cm).

Regarding the type of skin graft used, all cases underwent a conventional hin split-thickness skin graft, removed with a Blair knife, with small random perforations for drainage. Expansion of the grafts by means of a reticular apparatus (mesh graft) was not performed.

Lymphoscintigraphy (LFCG) was performed after a minimum interval of 12 months after the initial surgical treatment (skin grafts in the raw areas). We used the non-traumatized lower limb as a control.

To assess the lymphoscintigraphic findings, we used the Kleinhans Semiquantitative Index - KSI. We assessed the patterns of distribution, flow and path of lymphatic vessels, time to identify inguinal lymph nodes, and appearance, as well as the presence of collateralization, dermal reflux (DR), and the existence of a popliteal lymph node.

The Kleinhans Semiquantitative Index ranges from 0 to 45, values less than 10 being considered normal. The maximum time for lymph nodes to appear is 200 min, with a score of 8. The absence of uptake will receive a score of 9. For statistical analysis of these results, we used the Wilcoxon and Mann-Whitney tests. 

This study was approved by the Ethics in Research Committee of the Irmandade da Santa Casa de Misericórdia of São Paulo, being registered under number (CAAE) 54209416.4.0000.5479 and opinion 1.569,384. All patients signed an informed consent form.

## RESULTS

In the period from 2010 to 2016, 75 victims of degloving injuries with involvement of the lower limbs were treated, and of these, 18 met the inclusion criteria. In this group, 12 (66.7%) were males and six (33.3%) females. All patients had been run over by motor vehicles. The mean age observed was 28.11 years (12-48).

**Figure 1:**
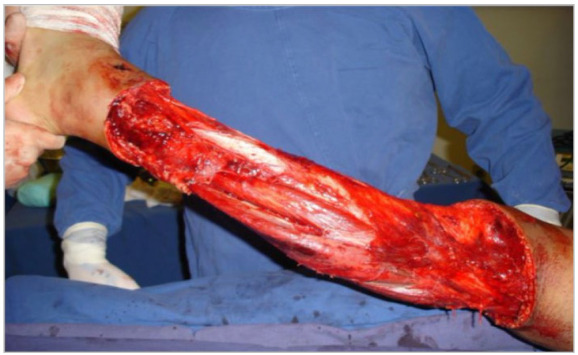
Case 1 - Circumferential degloving injury in the left leg, 9% ABS, vertical extension of approximately 35cm; admission.

**Figure 2:**
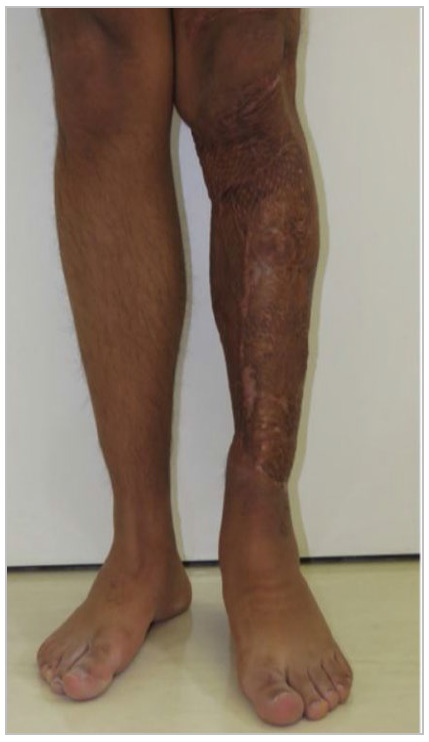
Case 1 - Circumferential degloving injury in the left leg, 9% ABS, vertical extension of approximately 35cm; 26 months after partial skin graft.

**Figure 3:**
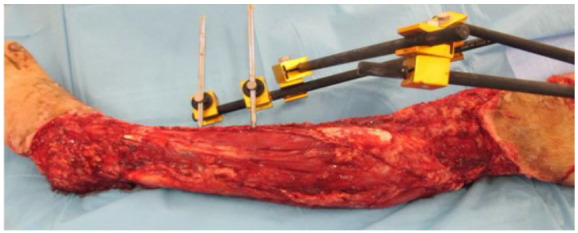
Case 2 - Circumferential degloving injury in left foot, ankle, leg, and left knee, 10% ABS, vertical extension of approximately 50cm; admission.

**Figure 4:**
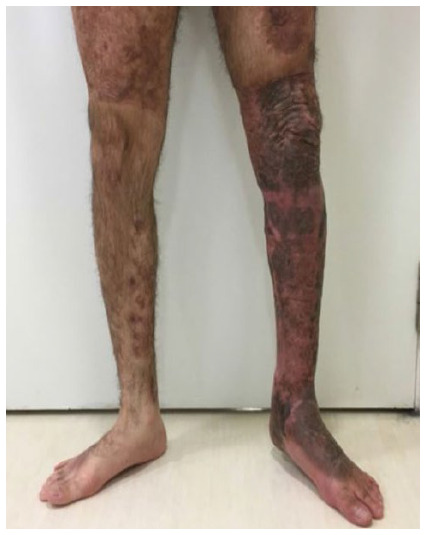
Case 2 - Circumferential degloving injury in left foot, ankle, leg and left knee, 10% ABS, vertical extension of approximately 50cm; 20 months after partial skin graft.

**Figure 5:**
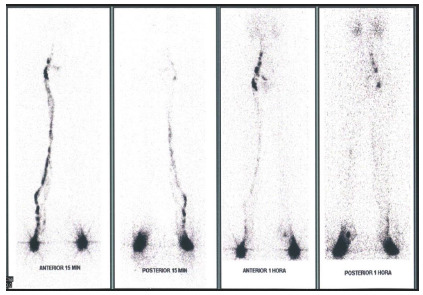
Lymphoscintigraphy of case 1.

**Figure 6:**
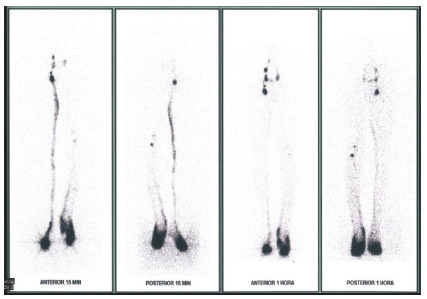
Lymphoscintigraphy of case 2.

The average affected body surface was 8.95% (5-15%). The mean vertical extension of the traumatized segment was 29.33cm (15-50cm) (Figures 1 to 6).

*• Time to appearance of inguinal lymph nodes: Right = 8 min / Left = 30 min.*

*• Left = Marked flow reduction, mild DR, reduced lymphatic vessels, reduced inguinal lymph nodes.*

*• KSI Right = 0.6 / Left = 20.75 .*

*• Time to appearance of inguinal lymph nodes: Right = 4 min / Left = 13 min.*

*• Left = Marked flow reduction, mild DR, reduced vessels, reduced inguinal lymph nodes, popliteal lymph node present.*

*• KSI Right = 0.37 / Left = 14.97.*

As for the affected side, the left corresponded to 55.6% of the cases (10), and the right, to the other eight patients. The time elapsed between treatment and lymphoscintigraphy was on average 22.55 months (13-31).

Regarding the occurrence of clinical manifestations in the traumatized limb (TL), these were present in 14 cases (77.7%), only edema being evidenced, observed from 12 to 18 months (according to reports and subjective assessment of patients). No local infection or thromboembolic events were observed, either in the TL or in the control limb (CL).

As for the classification of the International Society of Lymphology, we observed four Grade 0 cases, six Grade 1, and eight Grade 2 cases. There were no Grade 3 cases. 

All patients underwent the LFCG exam within the established protocol and without complications. Changes in the TL examination were present in 13 cases (72.22%) and absent in five. The LFCG exam was normal in all CLs.

The mean KSI observed in the TL was 8.32 (0.3-20.75), and we observed indexes >10 in nine patients. In CL the mean value was 0.58 (0.15-0.75) (Table 1 and Figures 7 and 8).

**Table 1:** Comparison between TL and CL lymphoscintigraphic exams.

	KSI TL	KSI CL	p-value
Average	8.32	0.58	0.001
Median	8.62	0.63	
95% CI	5.07-11.56	0.49-0.68	

Wilcoxon Test.

We observed five normal exams in the traumatized limbs, of which three did not present clinical manifestations. The other two had edema. In the group of patients who had altered lymphoscintigraphic exams (13), only one had no clinical manifestation. Table 2 shows the comparison of patients with normal and altered KSI exams.

**Table 2:** Comparison between normal and altered lymphoscintigraphic exams.

	KSI - TL	KSI - CL
Normal exam - 5 cases	0.63	0.55
Altered exam - 13 cases	11.27	0.6

Table 3 shows the comparative data of the KSI observed in TLs.

**Table 3:** Comparison between TL lymphoscintigraphic exams (KSI).

	Normal 5 cases	Altered 13 cases	p-value
Average	0.63	11.27	0.001
Median	0.75	11.85	
95% CI	0.27-0.98	8.19-14.36	

Mann-Whitney Test.

As for the occurrence of TL fractures, there were 11 cases. Table 4 brings the data referring to the findings of the LFCG exam. There were no cases of fractures in the CL.

**Table 4:** Relationship between LFCG results and fractures in the traumatized limb (TL).

	Normal LFCG	Altered LFCG
Fracture - Present	4	7
Fracture - Absent	1	6

## DISCUSSION

There has been an increase in the incidence of lymphedema, but unfortunately there is limited attention by the public and supplementary health systems, even in developed countries^21^ ^-^ ^23^.

Lymphedema is an inhibiting factor for healing. After trauma, there is the formation and opening of collateral lymphatic vessels, as well as the opening of connections between the superficial and deep systems^10.15,24,25^. 

Trauma rarely presents itself as an etiology for lymphedema, and there are few related publications. Lesser lesions, even if repetitive, present effective healing and vascular and lymphatic repair. For more extensive lesions, observations in the literature are inconsistent^26, 27^.

As mentioned, the occurrence of post-traumatic lymphedema is a rare and even unpredictable occurrence. The existing observations come from case reports, mostly or at most from small series. One must also consider the limitations for analyzes based on these publications, given the different etiologies involved. Cases have been reported after a few months to a few years. One could question whether there was an evaluation only from more evident edema cases, with a possible existence of undiagnosed cases in earlier periods.

LFCG is the most used test to assess the lymphatic system, as well as for suspected lymphedema, still considered the standard test. It is a non-invasive procedure and is associated with minimal discomfort. Its disadvantages are the use of ionizing radiation and limited anatomical resolution^28^ ^-^ ^30^. 

As for the LFCG findings, the presence of a popliteal lymph node is a sign of failure of lymphatic drainage through normal pathways. Dermal reflux is a sign of contrast extravasation due to hypertension in the proximal collectors, and contrast retention is a sign of overload and increased local hydrostatic pressure. Collateral circulation is also a pathological marker^31^. The Kleinhans’s index has a sensitivity of 97.4% and a specificity of 90.3%. It is considered reliable and is well associated with clinical conditions^32^ ^,^ ^33^.

As for the time to perform the LFCG, we did not find any reference to similar cases in the literature for comparison. We chose to establish a period of 12 months after the partial skin graft, believing that the edema related to the usual postoperative period would have already regressed.

We observed five normal LFCG exams in the TL, and three of these patients also did not present symptoms. In the other two cases, there was edema. We did not find infectious conditions or thromboembolic events. The possibility of the occurrence of clinical or LFCG alterations in a longer follow-up period is disputed.

We did not expect normal results in LFCG exams in TL. There are no similar cases in the literature that allow for comparisons. From an anatomical point of view, we can assume that there was a complete loss of conventional superficial lymphatic circulation pathways in the lower limbs secondary to circumferential and extensive degloving injury.

Although there are no specific studies in the literature, we believe that the fenestration of the skin layers used may mean extra damage to the residual dermis and greater difficulties for recanalization or maintenance of flow through the residual lymphatic vessels. 

split-thickness skin grafting is an unfavorable factor for the neoformation of lymphatic vessels, although this is an impression of case reports or small series. The available results show the absence of vessels in the segment directly below the grafted skin layers, which is a significant problem in cases such as the ones in this series, as they are grafts of greater length.

As for the existence of TL fractures, there was no association with severity or alterations in LFCG. In the 11 cases with fractures, four had normal exams. LFCG displayed alterations in six of the seven cases without fractures.

In our cases, all treatments for fractures were successful, without the occurrence of instability, lack of consolidation, or pseudarthrosis. All patients had open fractures with the possibility of bone coverage despite the soft tissue injury (Gustillo IIIa). More complex reconstructions with flaps (Gustillo IIIb) were not necessary, nor were associated vascular lesions requiring repair (Gustillo IIIc).

Sapienza et al.^33^ highlighted the identification of prominent lymphatic vessels or those with collaterals, as well as DR, in cases of edema after trauma or operations. The observation by Van Zanten et al.^18^ is that there can be recanalization or even regeneration in scar areas or after trauma. These authors evaluated 17 cases of patients with Gustillo IIIb open fractures. Factors such as inflammation and hypoxia can also be limiting for the neoformation of lymphatic vessels^34^ ^,^ ^35^. 

Lymphedema must be understood as a chronic, incurable, and slowly progressive disease, requiring lifelong treatment. The need for continuous care does not mean it cannot be effective or satisfactory^2^ ^,^ ^3^ ^,^ ^36^. In cases of treatment failures or difficulties, progressive skin changes, recurrent infections, progressive physical limitations, and psychosocial problems are observed^2^.

Considering the mechanism involved for the occurrence of degloving injuries, with transfer of significant amounts of energy after the contusion, in addition to the frequent association with fractures, we can assume limited lymphatic circulation regeneration after skin grafting, which in fact occurred in most of our cases. 

Prevention is directly related to clinical follow-up and active investigation of cases, which should already be stratified as higher or lower risk for early lymphedema. Importantly, LFCG can demonstrate lymphatic alterations before the existence of clinical alterations^9^.

There is still no definition regarding the moment to start treatment of mild or subclinical cases, as well as the best moment for the indication in the case of physiological operations, in the latter case considering the potential prophylactic effect. 

The number of early diagnoses should increase. While this does not occur, the identification of populations at higher risk, in need of intensive surveillance, is essential. Victims of lower limb degloving injury, especially circumferential injuries, are included in this group.

Due to the small number of cases that could meet the proposed inclusion criteria, in particular the occurrence of circumferential unilateral trauma with a preserved contralateral limb to allow comparison of the lymphoscintigraphic examination, we did not carry out a sample size calculation, nor other more elaborate statistical analyses.

Such a small group analysis is undoubtedly a significant limitation of the study. However, it demonstrates a high risk of complications and confirmation by an accessible complementary exam in more complex cases of this type of trauma to the lower limbs. These findings even led us to change the post-trauma follow-up routine in patients who suffered from degloving injuries in our service, which will allow for further analyzes and publications in the future.
